# Draft Genome Sequence of Pseudoalteromonas sp. Strain JC3

**DOI:** 10.1128/MRA.00212-21

**Published:** 2021-09-09

**Authors:** Margaret E. Rosario, Jacqueline Camm, Damian Cavanagh, David C. Rowley, David R. Nelson

**Affiliations:** a Department of Biomedical and Pharmaceutical Sciences, University of Rhode Island, Kingston, Rhode Island, USA; b Department of Cell and Molecular Biology, University of Rhode Island, Kingston, Rhode Island, USA; Portland State University

## Abstract

We report the draft genome sequence for Pseudoalteromonas sp. strain JC3, an isolate obtained from an aquaculture facility for whiteleg shrimp (Litopenaeus vannamei). The JC3 genome suggests multiple mechanisms for microbial interactions, including a type VI secretion system and potential for antibiotic production.

## ANNOUNCEMENT

Pseudoalteromonas is a genus of marine gammaproteobacteria with substantial capability for antibiotic production (1, 2). Recently, *Pseudoalteromonas* isolates have been suggested as potential probiotic bacteria to help combat acute hepatopancreatic necrosis disease (AHPND), an emerging and severe shrimp disease affecting aquaculture systems (3–5). Here, we provide details of the genome of a *Pseudoalteromonas* strain derived from a shrimp aquaculture facility to aid future investigations into the microbial and host interactions involving *Pseudoalteromonas* strains.

*Pseudoalteromonas* sp. strain JC3 was isolated from seawater of a whiteleg shrimp (Litopenaeus vannamei) culture purchased from Miami Aquaculture (Boynton Beach, FL). Seawater (100 μl) was spread onto YP30 agar plates (0.1% yeast, 0.5% peptone from meat, 3% Instant Ocean) and incubated at 25°C for 48 h. A yellow pigmented colony designated JC3 was iteratively inoculated onto YP30 agar and reisolated to ensure a pure culture.

*Pseudoalteromonas* sp. strain JC3 was grown in mLB30 media (Luria broth with 3% Instant Ocean) for 24 h at 25°C and 100 rpm. Genomic DNA was extracted using the Bio Basic molecular biology kit according to the manufacturer’s protocol. Genomic DNA was quantified using a Qubit fluorometer (Invitrogen) and sheared using a Covaris ultrasonicator. Libraries were prepared on an Apollo next-generation sequencing (NGS) library prep system using the PrepX DNA library kit (TaKaRa Bio) and run on an Agilent BioAnalyzer DNA high-sensitivity (HS) chip. Quantification was performed on all samples using quantitative PCR (qPCR) in a Roche LightCycler480 as described by the Roche KAPA Library Quantification Kit for Illumina Platforms technical data sheet (https://rochesequencingstore.com/wp-content/uploads/2017/10/KAPA-Lib-Quant-ILMN_9.17-IfU_1.pdf). Sequencing was performed at the Rhode Island Genomics and Sequencing Center using 2 × 250-bp paired-end sequencing on an Illumina MiSeq instrument. The total number of reads was 5,655,047 bp. Sequence trimming and quality control were completed using FastQC v1.0.0. Reads shorter than 64 bp were discarded. *De novo* assembly was performed using CLC Genomics Workbench v12.0.2, and the resulting contigs were processed using the CLC Microbial Genome Finishing module. The final draft assembly was estimated as 100% complete with 1.03% contamination using CheckM v1.0.18 (6) in KBase (7). The completed draft genome sequence is composed of 112 contigs (*N*_50_ contig length, 196,838 bp) with a total sequence length of 5,572,526 bp and an average G+C content of 43.1%. Rapid Annotations using Subsystems Technology (RAST) was utilized for gene annotation, which resulted in 5,021 coding sequences and 100 RNAs (8). The SEED viewer identified 354 subsystems containing 28% of the coding sequences (9). Default settings were used unless otherwise specified.

Assessment of the *Pseudoalteromonas* sp. strain JC3 draft genome sequence suggests multiple avenues for interacting with the surrounding microbial community and environment. BLASTp v12.11.0 (10) was used to search the nonredundant protein database at NCBI to identify genes for type II, IV, and VI secretion systems and a range of putative protein- and carbohydrate-degrading enzymes. Homologs of the *lodAB* (query coverage, 100%; E value, 0.0; amino acid identity, >99.5%) and *goxAB* (query coverage, 100%; E value, 0.0; amino acid identity, >99%) gene clusters found in Pseudoalteromonas flavipulchra JG1 and numerous bacterial groups (11) encoding marinocine (12) and glycine oxidase products (13), respectively, were also present. Analysis using the Antibiotics and Secondary Metabolite Analysis Shell (antiSMASH) (14) revealed 19 putative biosynthetic gene clusters, including one for the production of alterochromide/bromoalterochromide antibiotics (1).

### Data availability.

This whole-genome shotgun project has been deposited at DDBJ/ENA/GenBank under the accession number JAFEKQ000000000. The version described in this paper is the first version. The contigs are available at https://www.ncbi.nlm.nih.gov/Traces/wgs/JAFEKQ01?display=contigs. The raw reads can be found at the SRA under the accession number PRJNA699263.
